# Method for correcting respiratory artefacts in parallel-accelerated first-pass myocardial perfusion imaging

**DOI:** 10.1186/1532-429X-16-S1-P58

**Published:** 2014-01-16

**Authors:** Merlin Fair, Peter Gatehouse, Peter Drivas, Francisco Alpendurada, David Firmin

**Affiliations:** 1 Royal Brompton Hospital, London, UK; 2 Imperial College, London, UK

## Background

Myocardial first-pass perfusion (MPI) requires single-shot imaging of multiple slices per cycle. Breath-holding supports advanced high-resolution MPI methods, but tolerance to respiratory motion is desirable. Respiratory misregistration can induce aliasing artefacts by inaccurate coil sensitivity calibration, particularly at higher acceleration factors and during stress hyperpnea. Coil calibration can be adapted for respiratory motion, but autocalibration ("integrated") methods limit acceleration, and temporal coil-calibration methods may reduce SNR or cause temporal smoothing. We evaluated a motion-tracking modification of "prescan" parallel imaging specifically for MPI.

## Methods

To compare the conventional and new methods, rest MPI was acquired in 20 consented patients referred for late-enhancement imaging (0.1 mmol/kg GBCA). Patients were asked to breathe "slowly and deeply" during MPI to mimic potentially increased motion due to stress (Cartesian FLASH, 2.6 × 2.6 × 10 mm, GRAPPA rate 4, 3 SAX slices/cycle). The new method repeats coil prescans over the range of free-breathing motion ("Multiple Free-breathing Prescans", MFP) before acquiring MPI. For each reconstructed image, MFP aimed to use a prescan at the closest respiratory position. For this respiratory position-matched prescan selection, the MPI image was initially reconstructed using any of the prescans. The anterior chest wall location in this potentially artifactual image was compared with its location in every prescan, using a semi-automatic edge-detection algorithm. This enabled selection of the optimal coil prescan for each image of a final GRAPPA reconstruction (labeled MFP). The initial MPI image using a Single Conventional Prescan for its GRAPPA reconstruction (labeled SCP) was compared with MFP by randomised blinded scoring of parallel artefact (0 = none to 4 = non-diagnostic). In 5 further patients, MFP was compared with other coil calibrations (SCP, Integrated, Temporal) at increasing accelerations (R 2-6) by retrospective subsampling of full k-space MPI scans. The root-mean-square difference error (RMSE) over the FOV compared accelerated vs fully-sampled images.

## Results

**Figure 1:**
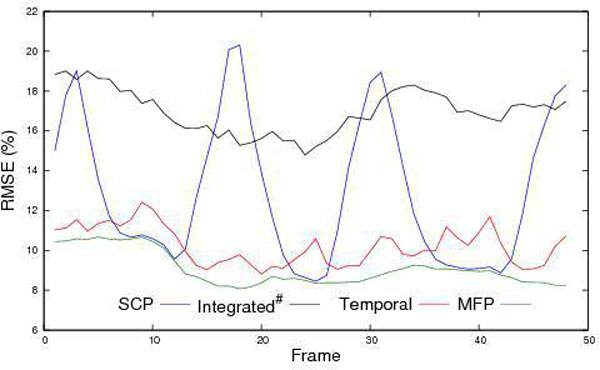
**Frame-by-frame RMSE of MPI series during deep respiration, using four coil-calibration methods**. RMSE measure of parallel artefact and noise was consistently small by MFP selecting optimum coil prescans for each MPI frame. (# Integrated method had outer-reduction-factor and number of reference lines chosen to give the same true acceleration factor as in the other methods, R = 4).

**Figure 2:**
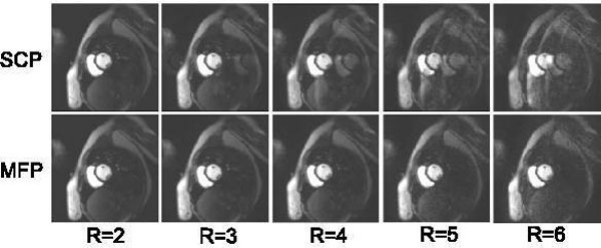
**Reconstruction at rates 2-6 (left-right) of a single MPI frame**. Coil position mismatch by respiration causes artefact (SCP, top row) which is corrected using the MFP method (bottom row).

Accelerated patient scans showed significantly (p < 0.02; n = 20) reduced artefact scores (MFP 1.15 ± 0.88 vs SCP 2.40 ± 1.31). Retrospective subsampling at R = 4 resulted in the lowest (p < 0.02, n = 5) mean RMSE by MFP 11.0 ± 1.3 vs Integrated 18.8 ± 3.8, SCP 13.6 ± 0.8, Temporal 11.8 ± 1.0 (Figure 1). Discussion: The bright chest-wall reliably supported position-detection of the anterior coil for prescan selection, making MFP apt for short-axis MPI; apart from prescan selection, MFP is a simple prescan reconstruction. Comparison against SCP is a basic test; further work might compare (or combine) with temporal methods, improve coil position matching and apply to faster methods (Figure 2).

## Conclusions

The MFP method by semi-automated image-based selection of a position-matched prescan reduces parallel imaging aliasing artefact in free-breathing MPI.

## Funding

Biomedical Research Unit, National Institute for Health Research.

